# Monitoring and orthogonal control of agrobacteria in *Nicotiana benthamiana* leaves

**DOI:** 10.1111/pbi.70056

**Published:** 2025-03-24

**Authors:** William Holdsworth, Zacharie LeBlanc, Sarah Moddejongen, Kaylee Moffitt, Christina Theodoropoulos, Robert E. Speight, Peter Waterhouse, Frank Sainsbury, James B. Behrendorff

**Affiliations:** ^1^ Centre for Agriculture and the Bioeconomy, School of Biology and Environmental Science Queensland University of Technology (QUT) Brisbane QLD Australia; ^2^ ARC Centre of Excellence in Synthetic Biology Queensland University of Technology (QUT) Brisbane QLD Australia; ^3^ Central Analytical Research Facility, Research Infrastructure Queensland University of Technology (QUT) Brisbane QLD Australia; ^4^ Advanced Engineering Biology Future Science Platform Commonwealth Scientific and Industrial Research Organisation (CSIRO) Dutton Park QLD Australia; ^5^ Centre for Cell Factories and Biopolymers, Institute for Biomedicine and Glycomics Griffith University Brisbane QLD Australia

**Keywords:** agrobacterium, transient transfection, synthetic biology, plant‐microbe interaction, *Nicotiana benthamiana*

Agrobacterium‐mediated transient transfection of *Nicotiana benthamiana* remains the most popular method for rapid synthesis of heterologous proteins in plants; yet relatively little is known about agrobacterial population stability, physiological state or plasmid maintenance following infiltration into *N. benthamiana* leaves. Developing a better understanding of post‐infiltration agrobacterium populations is important for designing new tools and strategies to exploit the agrobacterium–plant interaction using synthetic biology. In this study, we developed molecular tools and methods for monitoring and manipulating agrobacteria within leaf tissue. This capability may support the development of engineered agrobacteria for diverse applications such as reporting on plant physiology or synthesising additional metabolites while residing in the leaf, in parallel with plant metabolism.

*Nicotiana benthamiana* is a preferred host for heterologous protein expression due to innate hyper‐susceptibility to transfection and tolerance to infiltration of leaf tissue with agrobacteria (Bally *et al*., 2015), which transfer linear DNA (T‐DNA) to the plant nucleus via a type IV secretion system and a set of chaperone proteins encoded by the *vir* genes. Transferred DNA is transcriptionally active and can direct high levels of heterologous protein synthesis. Transfection occurs within the first day after infiltrating leaves with a suspension of agrobacteria, and the titre of plant‐synthesised heterologous protein typically peaks after 2–10 days before declining due to a combination of plant defence responses including RNA silencing and proteolysis (Grosse‐Holz *et al*., 2018). Though transfection occurs on the same day as infiltration, viable agrobacteria are still present in leaves when plants are harvested for downstream processing (Knödler *et al*., 2023).

To monitor agrobacteria post‐infiltration, *Agrobacterium fabrum* GV3101::pMP90 (previously known as *A. tumefaciens* GV3101::pMP90 (De Saeger *et al*., 2021)) was co‐transformed with a binary vector for transfecting plant cells with a T‐DNA encoding overexpression of a red fluorescent protein (pGGDNR_mCherry, where the pGGDNR vector is a modification of pCAMBIA1301) and a second plasmid encoding constitutive intracellular overexpression of a green fluorescent protein (GFP) within the agrobacterium (pSEVA431_pH‐tdGFP, where agrobacterial expression of pH‐tdGFP is controlled by a synthetic bacterial promoter, J23100) (Figure 1a). Predicted transcription and translation initiation rates for pH‐tdGFP under the control of different promoters used in this study are included in Table S1. Intracellular expression of pH‐tdGFP by *A. fabrum* was sufficient to enable visual monitoring of *A. fabrum* populations during *N. benthamiana* infiltration and transfection (Figure 1b, Figure S1). Further details of plasmid design are provided in Appendix S1.

**Figure 1:**
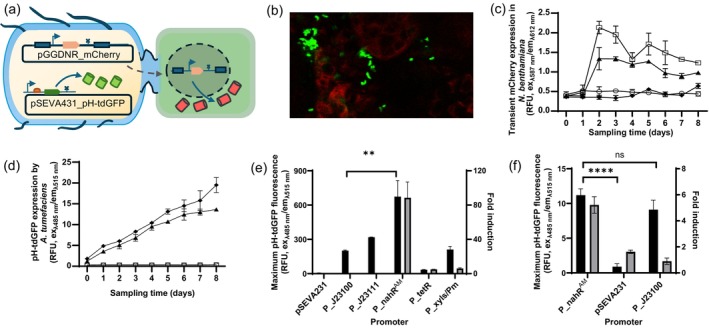
Monitoring and orthogonal control of *Agrobacterium fabrum* in *Nicotiana benthamiana* leaves. (a) Graphic description of an agrobacterium (in blue) bearing two plasmids: pSEVA431_pH‐tdGFP encoding intracellular overexpression of a green fluorescent protein, and pGGDNR_mCherry encoding a T‐DNA for overexpression of a red fluorescent protein in transfected plant cells (green). (b) Confocal microscopy image of transfected mesophyll cells expressing mCherry (red) and agrobacteria expressing pH‐tdGFP (green). (c, d) Fluorescence corresponding to mCherry (c) or pH‐tdGFP (d) was measured in extracts from leaves infiltrated with *A. fabrum* that was transformed with pSEVA431 (○), pSEVA431_ph‐tfGFP (◆), pGGDNR_mCherry (**□**) or co‐transformed with both pSEVA431_ph‐tfGFP and pGGDNR_mCherry (▲), *n* = 3 replicate infiltrations, mean ± standard deviation. (e) Expression of pH‐tdGFP regulated by different promoters in *A. fabrum* liquid cultures. Left axis: Maximum pH‐tdGFP fluorescence signal recorded during liquid cultivation (black bars). Right axis: Fold induction of inducible promoters P_nahR^AM^, P_tetR and P_xyls/Pm determined as the ratio of maximum fluorescence between induced and uninduced states (grey bars). *n* = 3 biological replicates, mean ± standard deviation, **P < 0.01 (unpaired *t*‐test). (f) Expression of pH‐tdGFP regulated by different promoters in *A. fabrum* within *N. benthamiana* leaves, 6 days post‐infiltration. Sodium salicylate (100 μM) was infiltrated into leaves on the third day after infiltration with *A. fabrum*. Left axis: Maximum pH‐tdGFP fluorescence signal recorded in leaf extracts (black bars). Right axis: Fold induction determined as the ratio of pH‐tdGFP signal detected between salicylate‐treated leaves and untreated leaves (grey bars). *n* = 3 replicate infiltrations, mean ± standard deviation, ****P < 0.0001 (unpaired *t*‐test).

Leaf tissue was sampled daily for 8 days post‐infiltration. Leaf discs (1 cm diameter) were homogenised in phosphate buffered saline with 0.1% (w/v) Triton X‐100 (3 μL extraction buffer per mg fresh sample weight), and green and red fluorescence signals corresponding to pH‐tdGFP and mCherry were measured in 10 μL samples of leaf homogenate (complete details of all experimental methods are provided in the Appendix S1). The titre of heterologous mCherry protein synthesised by transfected plant cells peaked on the second day post‐infiltration before steadily declining (Figure 1c); yet synthesis of pH‐tdGFP by *A. fabrum* increased linearly for 8 days (Figure 1d). Nucleic acid staining has previously been used to pre‐stain agrobacteria prior to infiltration and then directly visualise their attachment to plant cells (Simmons *et al*., 2012), but long‐term monitoring of agrobacteria was not possible with this method due to loss of fluorescence in the first 72 h post‐infiltration. The expression data presented in Figure 1d indicate that *A. fabrum* present in the leaf was not only viable but retained sufficient metabolic activity to support a linear increase in pH‐tdGFP expression. Furthermore, the plasmid encoding pH‐tdGFP overexpression was maintained by agrobacteria in the leaf for at least 8 days in the absence of antibiotic selection. Retention of the plasmid in the absence of selective pressure may be due to slow agrobacterial growth rates within leaf tissue limiting the opportunity for plasmid loss. The discovery that agrobacteria can overexpress heterologous proteins for more than a week after infiltrating leaf tissue opens the possibility of implementing additional genetic programmes in agrobacteria post‐infiltration.

Samples of *A. fabrum* were extracted from leaf tissue at 0, 2, 4, 6 and 8 days post‐infiltration and inoculated into a minimal defined culture medium to further evaluate agrobacterial viability and maintenance of the pH‐tdGFP expression plasmid. Culture media included rifampicin and gentamycin to minimise growth of non‐agrobacterial strains but excluded spectinomycin and kanamycin required for selection of pSEVA431_pH‐tdGFP and pGGDNR_mCherry plasmids. The maximum growth rate of *A. fabrum* after extraction from leaf tissue was 0.095 h^−1^ ± 0.028 (mean ± standard deviation) and there was no significant difference in maximum growth rate between untransformed *A. fabrum* or strains bearing pSEVA431_pH‐tdGFP, pGGDNR_mCherry or both plasmids (Figure S2). There was also no significant difference in lag phase (the time taken to reach maximum growth rate) between samples taken 2, 4, 6 or 8 days post‐infiltration (one‐way ANOVA), suggesting that agrobacteria have a comparable physiological state across this sampling period, characterised by very low growth rate but with sufficient metabolic activity to support heterologous protein expression. Only samples extracted and inoculated into liquid culture on day zero immediately post‐infiltration took significantly longer (one‐way ANOVA *P* < 0.0001) to adapt to growth in minimal defined liquid medium and reach the maximum growth rate (Figure S3). Expression of pH‐tdGFP continued in liquid cultures after extraction from leaves, and after 36 h of cultivation, there was no significant difference in pH‐tdGFP titre between samples extracted from leaves 2, 4, 6 or 8 days post‐infiltration (Figure S4). The same was observed for mCherry expression with strains transformed with the pGGDNR_mCherry plasmid (where mCherry expression was controlled by the cauliflower mosaic virus 35S promoter, which is known to drive low levels of expression in agrobacteria (Jacob *et al*., 2002)) (Figure S5). These data demonstrate retention of both plasmids for at least a week in the absence of selective pressure.

Three different inducible promoter systems were tested to evaluate whether transgene expression could be chemically induced in agrobacteria resident in *N. benthamiana* leaves. Salicylate‐inducible (Meyer *et al*., 2019) (P_nahR^AM^), anhydrotetracycline‐inducible (Meyer *et al*., 2019) (P_tetR) and 3‐hydroxybenzoate‐inducible (Martínez‐García *et al*., 2023) (P_xyls/Pm) promoters were coupled to the pH‐tdGFP reporter. Promoter performance was initially characterised in *A. fabrum* in liquid culture. The greatest dynamic range and total pH‐tdGFP signal were observed with the salicylate‐inducible promoter P_nahR^AM^ (Figure 1e and Figure S6), where the pH‐tdGFP signal was induced >80‐fold upon application of 100 μM sodium salicylate, and the total signal was more than double that observed with synthetic constitutive promoters P_J23100 and P_J23111. Expression from the P_xylS/Pm promoter (induced with 1 mM 3‐hydroxybenzoate) was comparable to the P_J23100 constitutive promoter, though high background expression was observed in the uninduced state (Figure S6). Weak anhydrotetracycline‐inducible expression was observed with P_tetR (Figure 1e and Figure S4). Agrobacteria in the *A. fabrum* C58 lineage frequently develop spontaneous resistance to tetracyclines (Luo and Farrand, 1999), which could be a potential cause of the minimal expression observed in P_tetR strains. The P_nahRAM promoter was selected for testing in plant infiltration experiments due to its high dynamic range.

*Agrobacterium fabrum* transformed with P_nahR^AM^ was infiltrated into *N. benthamiana* leaves. Three days post‐infiltration, a 100 μM solution of sodium salicylate was infiltrated into the same leaves, and pH‐tdGFP expression was monitored with daily sampling. Three days after salicylate treatment, expression of pH‐tdGFP had increased by more than fivefold (6 days after the initial infiltration of agrobacteria into *N. benthamiana* leaves) (Figure 1f). Inducible reporter protein expression was not observed in *N. benthamiana* leaves when using the anhydrotetracycline (P_tetR) or 3‐hydroxybenzoate (P_xylS/Pm)‐inducible promoters (Figure S7). Poor induction may be due in part to the catabolism of chemical inducers by the plant host and removal from leaves via vascular transport. The low induction responses observed relative to those seen in agrobacterial liquid cultures highlight the need for very strong inducible promoters with a high dynamic range, such as P_nahR^AM^, or promoters that are inducible via environmental signals rather than small molecules.

Salicylic acid is upregulated during a variety of plant stress responses, and exogenous application of salicylic acid is associated with increased pathogen defence in *N. benthamiana* (Jiang *et al*., 2021). Despite the possibility of enhanced pathogen defence, salicylate‐induced pH‐tdGFP synthesis by *A. fabrum* was comparable to that observed with the strong constitutive promoter P_J23100, and significantly more than the background signal from the pSEVA231 negative control (Figure 1f). Subjecting *N. benthamiana* to drought stress also induced a >2‐fold increase in pH‐tdGFP expression by *A. fabrum* bearing the P_nahR^AM^ plasmid (Figure S8).

*Agrobacterium fabrum* is capable of inducible and stable protein overexpression in leaves for several days post‐infiltration, beyond the typical sampling window for plant‐based transient protein expression. This opens new opportunities for engineering host–microbe interactions in leaf tissue, such as endowing agrobacteria with new functions as a sensor and reporter of host metabolism and product formation, or as a new compartment contributing metabolites or co‐products.

## Author contributions

WH, ZL and JBB contributed to all experiments, design, analysis and drafting the manuscript. KM and SM contributed to experiments. CT supported confocal microscopy. RES, PW and FS contributed to design and drafting the manuscript.

## Supporting information


**Appendix S1** Materials and methods.
**Table S1** Plasmid information.
**Figure S1** Agrobacterial pH‐tdGFP and plant‐based mCherry fluorescence.
**Figure S2** Growth rate of *A. fabrum* extracted from leaves.
**Figure S3** Lag phase of *A. fabrum* extracted from leaves.
**Figure S4** Expression of pH‐tdGFP by *A. fabrum* in liquid culture after extraction from *N. benthamiana* leaves.
**Figure S5** Expression of mCherry by *A. fabrum* in liquid culture after extraction from *N. benthamiana* leaves.
**Figure S6** Inducible expression of pH‐tdGFP by *A. fabrum* in liquid culture.
**Figure S7** Expression of pH‐tdGFP regulated by different promoters in *A. fabrum* within *N. benthamiana* leaves, six days post‐infiltration.
**Figure S8** Expression of pH‐tdGFP in *A. fabrum* within leaves of drought stressed *N. benthamiana*, seven days post‐infiltration.

## Data Availability

The data that supports the findings of this study are available in the supplementary material of this article.
