# Supplementary material for: Monitoring and orthogonal control of agrobacteria in Nicotiana benthamiana leaves
Source: Plant Biotechnol J. 2025 Mar 24;24(1):81–3. doi: 10.1111/pbi.70056 (PMC12854898; doi:10.1111/pbi.70056)
Supplement: Supplementary file 1 — Appendix S1 Materials and methods. Table S1 Plasmid information. Figure S1 Agrobacterial pH‐tdGFP and plant‐based mCherry fluorescence. Figure S2 Growth rate of A. fabrum extracted from leaves. Figure S3 Lag phase of A. fabrum extracted from leaves. Figure S4 Expression of pH‐tdGFP by A. fabrum in liquid culture after extraction from N. benthamiana leaves. Figure S5 Expression of mCherry by A. fabrum in liquid culture after extraction from N. benthamiana leaves. Figure S6 Inducible expression of pH‐tdGFP by A. fabrum in liquid culture. Figure S7 Expression of pH‐tdGFP regulated by different promoters in A. fabrum within N. benthamiana leaves, six days post‐infiltration. Figure S8 Expression of pH‐tdGFP in A. fabrum within leaves of drought stressed N. benthamiana, seven days post‐infiltration. [file PBI-24-81-s001.docx]

**Supporting Information**

Holdsworth *et al*. Monitoring and orthogonal control of agrobacteria in *Nicotiana benthamiana* leaves

**Appendix S1 Materials and Methods**

**Plant seeds and bacterial strains**

Seeds for *Nicotiana benthamiana* were obtained from the laboratory of Prof. Peter Waterhouse, Queensland University of Technology. Molecular cloning and plasmid maintenance was performed in *Escherichia coli* Top10 (Invitrogen, ThermoFisher Scientific catalogue number C404003). *Agrobacterium fabrum* GV3101::pMP90 (previously known as *Agrobacterium tumefaciens* GV3101::pMP90) was obtained from the laboratory of Prof. Peter Waterhouse, Queensland University of Technology.

**Plasmids**

Plasmids described in Supporting Table S1 were assembled using the general principles of Gibson assembly^1^, or synthesised commercially by Twist Bioscience. Predicted transcription initiation rates^2^ and translation initiation rates^3,4^ were predicted using the denovodna.com Promoter Calculator and RBS Calculator, respectively.

**Supporting Table S1**

| **Plasmid name** | **Vector details** | **Description** | **Bacterial transcription initiation rate** | ***A. fabrum* translation initiation rate** |
| --- | --- | --- | --- | --- |
| pSEVA_pH-tdGFP | pSEVA431^5^, spectinomycin resistance | pH-td_GFP^6^ expression regulated by the J23100 promoter from the Anderson Library^7^ of synthetic promoters. | 518 | 1498 |
| pGGDNR_mCherry | pCAMBIA1301, kanamycin resistance | T-DNA region bearing mCherry^8^ coding sequence under the control of the CaMV35S promoter. | N/A | N/A |
| pSEVA231 | pSEVA231^5^, kanamycin resistance | No fluorescent protein coding sequence | N/A | N/A |
| P_J23100 | pSEVA231, kanamycin resistance | pH-tdGFP expression regulated by the J23100 promoter from the Anderson Library of synthetic promoters. | 518 | 1498 |
| P_J23111 | pSEVA231, kanamycin resistance | ph-tdGFP expression regulated by the J23111 promoter from the Anderson Library of synthetic promoters. | 518 | 1498 |
| P_nahR^AM^ | pSEVA231, kanamycin resistance | pH-tdGFP expression regulated by the nahR^AM^ salicylate inducible promoter^9^. | 2558 | 1137 |
| P_xylS/Pm | pSEVA231, kanamycin resistance | pH-tdGFP expression regulated by the xylS/Pm 3-hydroxybenzoate inducible promoter system^10^. | 490 | 2668 |
| P_tetR | pSEVA231, kanamycin resistance | pH-tdGFP expression regulated by the tetR tetracycline inducible promoter^9^. | 375 | 1137 |

**Plant infiltration, sampling, and measurement of pH-tdGFP and mCherry fluorescent proteins**

Agrobacteria were inoculated from glycerol stocks into 50 mL of YEP medium (10 g yeast extract, 10 g peptone, and 5 g sodium chloride per litre) supplemented with rifampicin (25 µg/mL), gentamycin (30 µg/mL), and either kanamycin (50 µg/mL for all plasmids except pSEVA_pH-tdGFP) or spectinomycin (100 µg/mL, for pSEVA_pH-tdGFP) and grown at 28 °C with agitation at 200 RPM for approximately 24-36 hours they reached an OD_600 nm_ of 1.3-1.8. Cultures were washed and diluted in infiltration buffer (10 mM 2-morpholinoethanesulfonic acid, 10 mM magnesium chloride, pH 5.7) to an OD_600 nm_ of 0.5. Agrobacteria suspensions were infiltrated into the abaxial surface of *N. benthamiana* leaves with a needleless syringe.

Leaf discs were sampled daily with a 1 cm cork borer and weighed on an analytical balance. Leaf discs were homogenised in three volumes of extraction buffer (phosphate buffered saline plus 0.1 % (v/v) Triton X-100) per mg fresh weight (e.g. 30 µl extraction buffer per 10 mg leaf disc). Leaf discs were homogenised by grinding with a micropestle in microcentrifuge tubes. Homogenate samples (10 µL) were transferred to a white 384-well microtitre plate and fluorescence was measured in a Varioskan Lux multimode plate reader (ThermoFisher Scientific) with the following settings. Fluorescence corresponeding to pH-tdGFP (excitation wavelength 485 nm, emission wavelength 515 nm) and mCherry (excitation wavelength 587 nm, emission wavelength 612 nm) were recorded.

Agrobacteria extracted from leaf homogenate (1 µL) were inoculated into 99 µL of a modified M9 medium^11^ (M9++) supplemented with Studier’s trace metals^12^, casamino acids (0.2 %, w/v), thiamine (1 mM), rifampicin (25 µg/mL), and gentamycin (30 µg/mL). Cultures were incubated in a flat-bottomed 96-well microtitre plate sealed with a Breathe-Easy membrane (Merck cat. no. Z380059) and incubated at 28 °C with shaking in a Varioskan Lux multimode plate reader (ThermoFisher Scientific). Optical density (OD600 nm), pH-tdGFP fluorescence (excitation 485 nm, emission 515 nm), and mCherry fluorescence (excitation 587 nm, emission 612 nm) were recorded every 20 minutes for 48 h.

**Confocal microscopy**

*N. benthamiana* leaves were infiltrated with a solution of *A. fabrum* co-transformed with pSEVA_pH-tdGFP and pGGDNR-mCherry. Leaf discs were sampled two days post-infiltration using a 1 cm cork borer. Leaf discs were submerged in 4% (w/v) para-formaldehyde solution for 40 minutes to allow for complete fixation of the leaf tissue. Samples were washed twice in 1 x phosphate buffered saline (PBS) and stored submerged in 1 mL of PBS at 4 °C until imaging. On the day of Imaging, plant tissue was mounted on microscope slides and cover slipped with PBS to provide moisture.

Samples were analysed using a HCX PL APO CS 40 x 1.25 OIL UV objective on a Confocal Laser Scanning Microscope (Leica TCS SP5 CLSM; Leica Microsystems, Mannheim, Germany). Two channels were activated to capture the GFP-expressing bacteria (500nm - 530nm), and mCherry (600nm - 620nm).

**Characterisation of inducible promoters in agrobacteria liquid culture**

*A. fabrum* was transformed with plasmids pSEVA231, P_J23100, P_J23111, P_nahR^AM^, P_xylS/Pm, or P_tetR. Overnight liquid cultures were cultivated in 1 mL YEP medium supplemented with rifampicin (25 µg/mL), gentamycin (30 µg/mL), and kanamycin (50 µg/mL) at 28 °C with shaking at 600 rpm in a Vortemp microplate orbital shaker. Overnight starter cultures were inoculated 1 in 100 into 100 µL of M9++ medium including rifampicin, gentamycin, and kanamycin. Cultures inoculated with *A. fabrum* P_nahRAM, P_xylS/Pm, or P_tetR also included their corresponding inducer (100 µM sodium salicylate, 1 mM 3-hydroxybenzoate, or 0.2 µM anhydrotetracycline, respectively). Cultures were incubated in a flat-bottomed 96-well microtitre plate sealed with a Breathe-Easy membrane (Merck cat. no. Z380059) and incubated at 28 °C with shaking in a Varioskan Lux multimode plate reader (ThermoFisher Scientific). Optical density (OD600 nm), pH-tdGFP fluorescence (excitation 485 nm, emission 515 nm) were recorded every 20 minutes for 48 h.

**Characterisation of inducible promoters in agrobacteria infiltrated into *N. benthamiana* leaves**

*A. fabrum* bearing plasmids pSEVA231, P_J23100, P_J23111, P_nahR^AM^, P_xylS/Pm, or P_tetR were inoculated from glycerol stocks into 50 mL of YEP medium (10 g yeast extract, 10 g peptone, and 5 g sodium chloride per litre) supplemented with rifampicin (25 µg/mL), gentamycin (30 µg/mL), and kanamycin (50 µg/mL) and grown at 28 °C with agitation at 200 RPM for approximately 24-36 hours they reached an OD_600 nm_ of 1.3-1.8. Cultures were washed and diluted in infiltration buffer (10 mM 2-morpholinoethanesulfonic acid, 10 mM magnesium chloride, pH 5.7) to an OD_600 nm_ of 0.5. Agrobacteria suspensions were infiltrated into the abaxial surface of *N. benthamiana* leaves with a needleless syringe.

Three days post-infiltration, leaves were reinfiltrated with a solution of 100 µm sodium salicylate, 1 mM 3-hydroxybenzoate, or 0.2 µM anhydrotetracycline. Replicate leaves infiltrated with agrobacteria where no inducer was infiltrated were included as uninduced control samples. Leaf discs were sampled daily with a 1 cm cork borer and weighed on an analytical balance. Leaf discs were homogenised in three volumes of extraction buffer (phosphate buffered saline plus 0.1 % (v/v) Triton X-100) per mg fresh weight (e.g. 30 µl extraction buffer per 10 mg leaf disc). Leaf discs were homogenised by grinding with a micropestle in microcentrifuge tubes. Homogenate samples (10 µL) were transferred to a white 384-well microtitre plate and fluorescence was measured in a Varioskan Lux multimode plate reader (ThermoFisher Scientific) with the following settings. Fluorescence corresponding to pH-tdGFP (excitation wavelength 485 nm, emission wavelength 515 nm) and mCherry (excitation wavelength 587 nm, emission wavelength 612 nm) were recorded.

***N. benthamiana* drought stress as an inducer of the P_nahRAM promoter in *A. fabrum***

*Nicotiana benthamiana* plants were maintained in a hydrated state prior to agroinfiltration. *A. fabrum* bearing plasmids pSEVA231, P_J23100, and P_nahR^AM^, were prepared for infiltration as described above in *Characterisation of inducible promoters in agrobacteria infiltrated into N. benthamiana leaves*. The pSEVA231, P_J23100, and P_nahR^AM^ strains were infiltrated as three distinct spots within a single leaf, and this was repeated in three leaves per plant. From the day of agroinfiltration, six plants were deprived of water for four days to induce drought stress while the other half were maintained in a hydrated state. On the fourth day post-infiltration, the drought stressed plants were re-hydrated. Three drought-stressed plants were infiltrated with 100 µM sodium salicylate to provide a positive control for induction of pH-tdGFP expression by salicylate in drought-stressed plants. Three days after infiltration with sodium salicylate, leaf discs were harvested and pH-tdGFP was analysed as described above. GFP signals from P_nahRAM-infiltrated leaf extracts were normalised to GFP signals from P_J23100 extracts from within the same leaf, to account for inter-plant variability and possible direct impacts of drought treatment on agrobacterial protein expression.

**
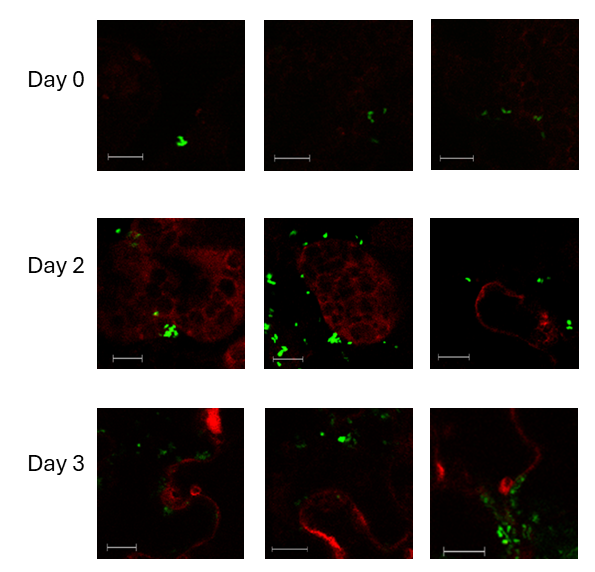
**

**Supporting Figure S1 Agrobacterial pH-tdGFP and plant-based mCherry fluorescence.** *A. fabrum* co-transformed with both pSEVA431_pH-tdGFP and pGGDNR_mCherry were infiltrated into *N. benthamiana* leaf tissue and fluorescence signals corresponding to pH-tdGFP and mCherry were monitored *via* confocal microscopy on days 0, 2, and 3 post-infiltration. Scale bar = 10 µm. Images from three replicate leaf infiltrations are arranged in each row.


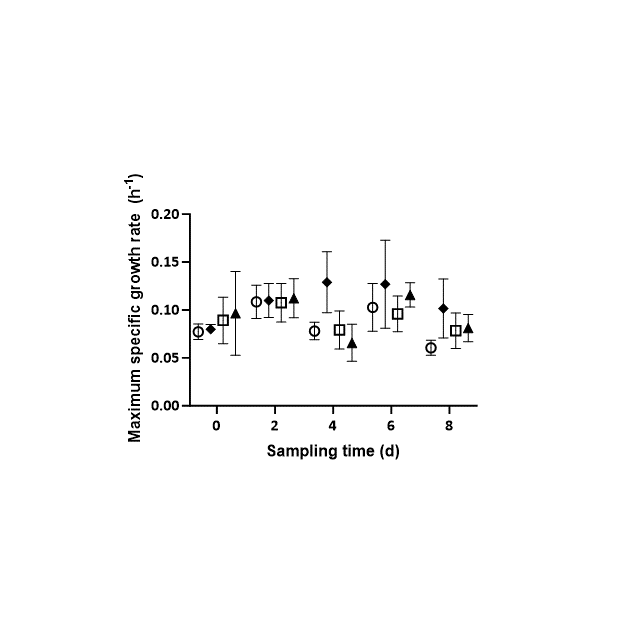


**Supporting Figure S2 Growth rate of *A. fabrum* extracted from leaves.** *A. fabrum* were extracted from leaf tissue 0, 2, 4, 6, and 8 days post-infiltration and inoculated into liquid culture and grown at 28 °C, monitoring the optical density for 48 h, and the maximum specific growth rate was calculated. *A. fabrum* strains were transformed with pSEVA431 (○), pSEVA431_pH-tdGFP (◆), pGGDNR_mCherry (**□**), or co-transformed with both pSEVA431_pH-tdGFP and pGGDNR_mCherry (▲), n = 3 replicate infiltrations, mean ± standard deviation.


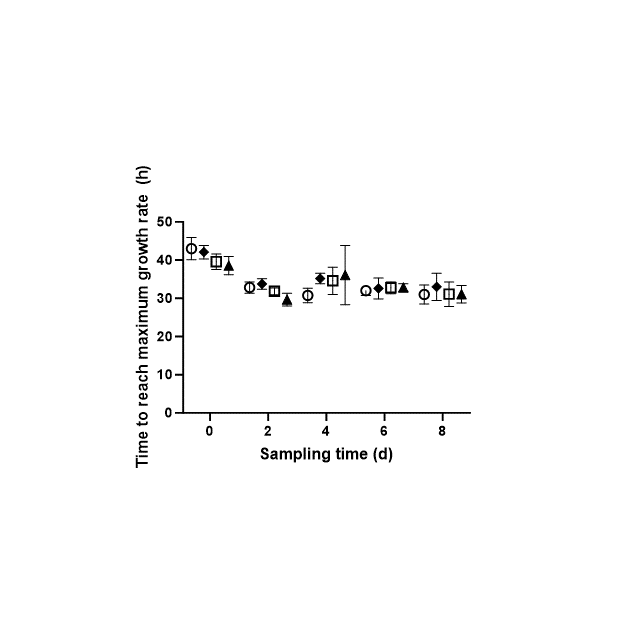


**Supporting Figure S3 Lag phase of *A. fabrum* extracted from leaves.** *A. fabrum* were extracted from leaf tissue 0, 2, 4, 6, and 8 days post-infiltration and inoculated into liquid culture and grown at 28 °C, monitoring the optical density for 48 h, and the time taken for cultures to reach the calculated maximum specific growth rate was determined. *A. fabrum* strains were transformed with pSEVA431 (○), pSEVA431_pH-tdGFP (◆), pGGDNR_mCherry (**□**), or co-transformed with both pSEVA431_pH-tdGFP and pGGDNR_mCherry (▲), n = 3 replicate infiltrations, mean ± standard deviation.

**Supporting Figure S4 Expression of pH-tdGFP by *A. fabrum* in liquid culture after extraction from *N. benthamiana* leaves.** *A. fabrum* were extracted from leaf tissue 0, 2, 4, 6, and 8 days post-infiltration and inoculated into liquid culture and grown at 28 °C. Green fluorescence corresponding to pH-tdGFP was monitored and the fluorescence signal 36 h after inoculation into liquid culture is reported for *A. fabrum* strains transformed with pSEVA431 (○), pSEVA431_pH-tdGFP (◆), pGGDNR_mCherry (**□**), or co-transformed with both pSEVA431_pH-tdGFP and pGGDNR_mCherry (▲), n = 3 replicate infiltrations, mean ± standard deviation.

**Supporting Figure S5 Expression of mCherry by *A. fabrum* in liquid culture after extraction from *N. benthamiana* leaves.** *A. fabrum* were extracted from leaf tissue 0, 2, 4, 6, and 8 days post-infiltration and inoculated into liquid culture and grown at 28 °C. Red fluorescence corresponding to mCherry was monitored and the fluorescence signal 36 h after inoculation into liquid culture is reported for *A. fabrum* strains transformed with pSEVA431 (○), pSEVA431_ph-tfGFP (◆), pGGDNR_mCherry (**□**), or co-transformed with both pSEVA431_ph-tfGFP and pGGDNR_mCherry (▲), n = 3 replicate infiltrations, mean ± standard deviation.

Time (h)

Time (h)

Time(h)

Time (h)

GFP fluorescence per unit biomass

(RFU /OD_600 nm_)

(A)

(B)

(C)

(D)

**Supporting Figure S6 Inducible expression of pH-tdGFP by *A. fabrum* in liquid culture.** *A. fabrum* transformed with different inducible promoter reporter plasmids were grown in liquid culture and the pH-tdGFP signal per unit biomass was measured. (A) P_nahRAM with (●) or without (○) 100 µM sodium salicylate. (B) P_xylS/Pm with (●) or without (○) 1 mM 3-hydroxybenzoate. (C) P_tetR with (●) or without (○) 0.2 µM anhydrotetracycline. Inset: zoomed view with adjusted x-axis. (D) P_J23100 (▲) or P_J23111 (△) constitutive expression plasmids, and pSEVA231 (○). n = 3 biological replicates, mean ± standard deviation.


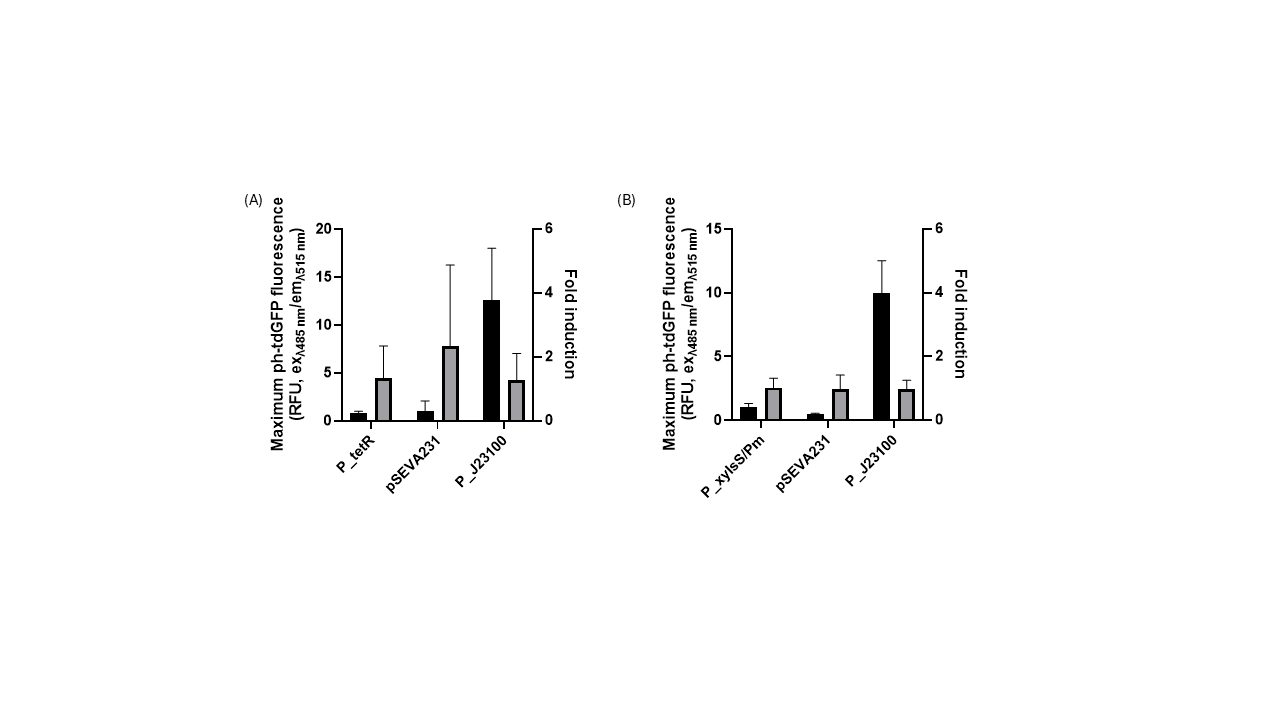


**Supporting Figure S7 Expression of pH-tdGFP regulated by different promoters in *A. fabrum* within *N. benthamiana* leaves, six days post-infiltration.** (A) Anhydrotetracycline (0.2 µM) was infiltrated into leaves on the third day after infiltration with *A. fabrum* bearing P_tetR, pSEVA231, or P_J23100. (B) 3-hydroxybenzoate (1 mM) was infiltrated into leaves on the third day after infiltration with *A. fabrum* bearing P_tetR, pSEVA231, or P_J23100. Left axis: maximum pH-tdGFP fluorescence signal recorded in leaf extracts (black bars). Right axis: fold induction determined as the ratio pH-tdGFP signal detected between inducer-treated and untreated leaves (grey bars). n = 3 replicate infiltrations, mean ± standard deviation.

**Supporting Figure S8 Expression of pH-tdGFP in *A. fabrum* within leaves of drought stressed *N. benthamiana*, seven days post-infiltration.** *A. fabrum* bearing P_nahR^AM^, pSEVA231, or P_J23100 was infiltrated *N. benthamiana* leaves, and post infiltration plants were either maintained in a hydrated state or subjected to four days of drought stress. On the fourth day after infiltration with *A. fabrum* drought stressed plants were rehydrated and three plants were infiltrated with 100 µM sodium salicylate (+ sal) while the others were not (- sal). Three days after rehydration (and seven days post-infiltration with agrobacteria), leaf discs were sampled and pH-tdGFP signal in leaf extracts was measured. GFP signals from P_nahR^AM^ extracts were normalised to the GFP signal from P_J23100 extracts from the same leaf. n = 9 replicate infiltrations (n = 3 plants per condition with 3 infiltrated leaves per plant), mean ± standard deviation. * = P < 0.05, ** = P < 0.01, Student’s *t*-test.

**Supplementary References**

1. Gibson, D. G. *et al.* Enzymatic assembly of DNA molecules up to several hundred kilobases. *Nat. Methods* **6**, 343–345 (2009).

2. LaFleur, T. L., Hossain, A. & Salis, H. M. Automated model-predictive design of synthetic promoters to control transcriptional profiles in bacteria. *Nat. Commun.* **13**, 5159 (2022).

3. Salis, H. M., Mirsky, E. A. & Voigt, C. A. Automated design of synthetic ribosome binding sites to control protein expression. *Nat. Biotechnol.* **27**, 946–950 (2009).

4. Espah Borujeni, A. & Salis, H. M. Translation Initiation is Controlled by RNA Folding Kinetics via a Ribosome Drafting Mechanism. *J. Am. Chem. Soc.* **138**, 7016–7023 (2016).

5. Martínez-García, E. *et al.* SEVA 4.0: an update of the Standard European Vector Architecture database for advanced analysis and programming of bacterial phenotypes. *Nucleic Acids Res.* **51**, D1558–D1567 (2023).

6. Roberts, T. M. *et al.* Identification and Characterisation of a pH-stable GFP. *Sci. Rep.* **6**, 28166 (2016).

7. Promoters/Catalog/Anderson - parts.igem.org. https://parts.igem.org/Promoters/Catalog/Anderson.

8. Shaner, N. C. *et al.* Improved monomeric red, orange and yellow fluorescent proteins derived from Discosoma sp. red fluorescent protein. *Nat. Biotechnol.* **22**, 1567–1572 (2004).

9. Meyer, A. J., Segall-Shapiro, T. H., Glassey, E., Zhang, J. & Voigt, C. A. Escherichia coli “Marionette” strains with 12 highly optimized small-molecule sensors. *Nat. Chem. Biol.* **15**, 196–204 (2019).

10. Gawin, A., Valla, S. & Brautaset, T. The XylS/Pm regulator/promoter system and its use in fundamental studies of bacterial gene expression, recombinant protein production and metabolic engineering. *Microb. Biotechnol.* **10**, 702–718 (2017).

11. M9 minimal medium (standard). *Cold Spring Harb. Protoc.* **2010**, pdb.rec12295 (2010).

12. Studier, F. W. Protein production by auto-induction in high-density shaking cultures. *Protein Expr. Purif.* **41**, 207–234 (2005).
